# Case report: Post-therapeutic laryngeal carcinoma patient possessing a high ratio of aneuploid CTECs to CTCs rapidly developed *de novo* malignancy in pancreas

**DOI:** 10.3389/fonc.2022.981907

**Published:** 2022-09-12

**Authors:** Jiaoping Mi, Fang Yang, Jiani Liu, Mingyang Liu, Alexander Y. Lin, Daisy Dandan Wang, Peter Ping Lin, Qi Zeng

**Affiliations:** ^1^ Department of Otolaryngology-Head and Neck Surgery, The Fifth Affiliated Hospital of Sun Yat-Sen University, Zhuhai, China; ^2^ Cancer Center, The Fifth Affiliated Hospital of Sun Yat-Sen University, Zhuhai, China; ^3^ State Key Laboratory of Molecular Oncology, National Cancer Center/National Clinical Research Center for Cancer/Cancer Hospital, Chinese Academy of Medical Sciences and Peking Union Medical College, Beijing, China; ^4^ Department of Oncology, Cytelligen, San Diego, CA, United States

**Keywords:** MRD, prediction of cancer occurrence, liquid biopsy, aneuploid CTCs and CTECs, SE-iFISH

## Abstract

Effectively evaluating therapeutic efficacy, detecting minimal residual disease (MRD) after therapy completion, and predicting early occurrence of malignancy in cancer patients remain as unmet imperative clinical demands. This article presents a case of a laryngeal carcinoma patient who had a surgical resection and complete post-operative chemoradiotherapy in combination with the targeted therapy, then rapidly developed pancreatic adenocarcinoma. Detected by SE-iFISH, the patient had a substantial amount of 107 non-hematological aneuploid circulating rare cells including 14 circulating tumor cells (CTCs, CD31^-^/CD45^-^) and 93 circulating tumor endothelial cells (CTECs, CD31^+^/CD45^-^) with a high ratio of CTECs/CTCs > 5 upon finishing post-surgical combination regimens. Positive detection of those aneuploid non-hematological circulating rare cells was five months prior to subsequent plasma CA19-9 increasing and ten months before the *de novo* pancreatic cancer was diagnosed by medical imaging modalities. Besides previously reported clinical utilities of co-detection of aneuploid CD31^-^ CTCs and CD31^+^ CTECs in real-time evaluation of therapeutic efficacy, longitudinal monitoring of emerging treatment resistance and adequate detection of MRD, a large cohort study is necessary to further investigate whether, and how, a high ratio of MRD CTECs to CTCs may function as an appropriate index forecasting either occurrence or metastatic distant recurrence of malignancy in post-therapeutic cancer patients.

## Introduction

Effectively evaluating therapeutic efficacy and predicting cancer patients possessing a high risk of malignancy remain highly challenging. Current clinical strategies to detect cancer occurrence mainly rely on medical imaging modalities, including computed tomography (CT) to show the location, anatomic shape, and size of a lesion; magnetic resonance imaging (MRI) that uses strong magnets to create cross-section images inside the body; and positron emission tomography (PET) to identify an increased glucose consumption spot of neoplasm. However, the hurdle of conventional clinical imaging is its limitation to detect only visible-sized tumors. Medical imaging detection of invisible minimal residual disease (MRD), which are a small number of cancer cells remaining in the body after treatment (1), has not yet been achieved.

The association of quantified circulating tumor cells (CTCs) with cancer occurrence has been reported elsewhere (2–5). Recent CTC studies have indicated that phenotypic and karyotypic variations in highly heterogeneous CTCs constantly occur throughout tumor progression and therapy (6). Conventional EpCAM and cytokeratin-dependent CTC enumeration alone is no longer sufficient to corelate the full spectrum of CTC characteristics with distinct clinical utilities (7).

Neoangiogenesis, a hallmark of cancers, is essential for tumorigenesis, progression, and cancer metastasis (8). A majority of endothelial cells (ECs) in tumor vasculatures are tumor endothelial cells (TECs), showing aneuploidy of chromosomes and phenotypic expression of CD31 (platelet endothelial cell adhesion molecule-1, PECAM-1) (9). TECs are predominately derived from the endothelialization of cancer cells and cancerization of ECs in the hypoxic tumor microenvironment (TME) (10). Contribution of diverse subpopulations of TECs to tumor progression (11), patients’ survival, VEGF blockade, and regulating immune surveillance has recently been described (12). Similar to CTCs, TECs also shed from tumor vasculatures into peripheral blood and turn into CD31^+^ aneuploid circulating TECs, known as CTECs (10, 13, 14). The clinical significance of CTECs, particularly PD-L1^+^ (15), the stemness marker CD44^+^ (16), and EpCAM^+^/Vimentin^+^ CTECs (17), in multiple types of cancers has been recently addressed (18–20). CTCs and CTECs are a unique pair of cellular circulating tumor biomarkers and have cross-talk and functional interplay in cancer patients (6, 10).

A novel strategy integrating subtraction enrichment (SE) and immunostaining-fluorescence *in situ* hybridization (SE-iFISH) has recently been reported to effectively co-detect and molecularly characterize aneuploid CTCs and CTECs (6, 13, 21). In contrast to conventional CTC detection methods, the EpCAM-independent subtraction enrichment (SE) strategy is able to enrich heterogeneously sized non-hematologic circulating rare cells, regardless of cell surface protein expression, followed by a comprehensive *in situ* phenotypic and karyotypic molecular characterization as well as categorization of enriched circulating rare cells (13, 21–23).

In this report, we present a case showing that a post-surgical laryngocarcinoma patient, after receiving a complete chemoradiotherapy, still showed a large quantity of CTCs and CTECs with a substantially high ratio of CTECs/CTCs > 5. Positive detection of CTCs and CTECs occurred ten months prior to the diagnosed pancreatic cancer. This case highlights potential unique clinical utilities of the co-detection of aneuploid CD31^-^ CTCs and CD31^+^ CTECs for adequate evaluation of treatment outcome, detection of MRD, as well as prediction of early occurrence of new primary lesion in post-therapeutic cancer patients.

## Case presentation

**Figure 1:**
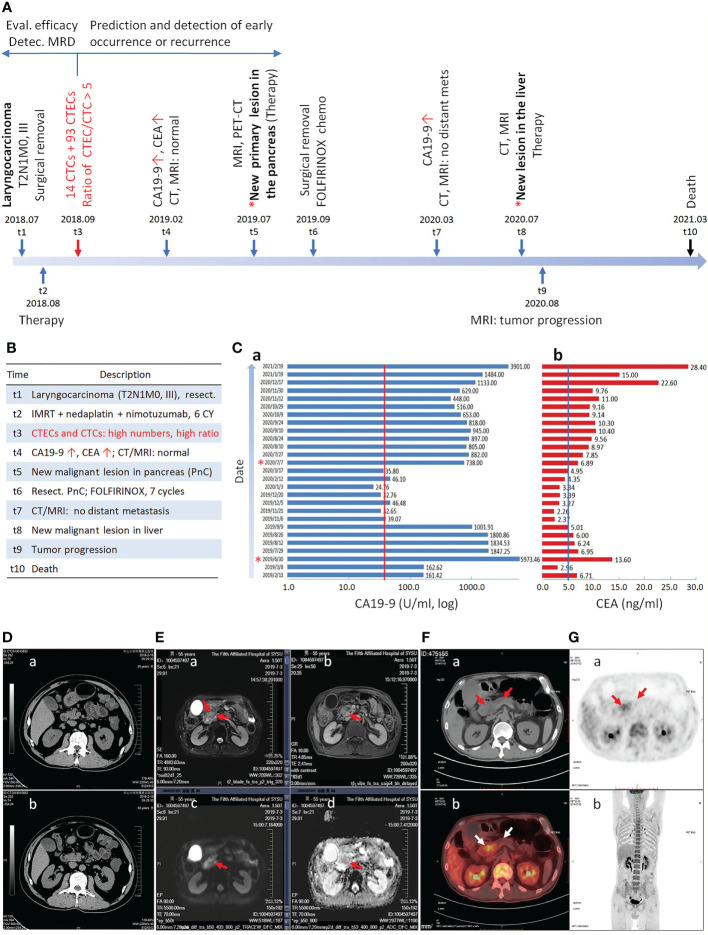
Longitudinal progression of the cancer patient. **(A)** Timeline of diagnosis and treatment of the patient at different time intervals is illustrated. The primary laryngocarcinoma is diagnosed at t1, followed by detection of CTCs and CTECs at t3. A new malignant lesion in the pancreas is confirmed at t5. Detection of CTCs and CTECs at the end of therapy could effectively evaluate therapeutic efficacy, detect MRD, and predict early occurrence of malignancy. **(B)** Description of each time point from t1 to t10. **(C)** Continuous monitoring of CA19-9 and CEA. The patient has the highest concentration of CA19-9 at t5. The cut-off values of CA19-9 (37 U/ml) and CEA (5 ng/ml) are indicated by red and blue line, respectively. **(D–G)** Medical imaging. **(Da, b)** Abdominal CT scan shows no abnormality in pancreas. **(Ea–d)** Epigastric MRI, CE-MRI, MRCP and DWI indicate the metastatic pancreatic head carcinoma. **(Ea)** A space-occupying lesion in pancreas T2WI (red arrows). **(Eb)** An increase contrast signal in a space-occupying lesion in pancreas T1WI (red arrow). **(Ec)** A strong signal in a space-occupying lesion in pancreas T1WI by DWI (red arrow). **(Ed)** ADC imaging shows decrease signal in a space-occupying lesion in pancreas T1WI (red arrow). **(F, G)** A pancreatic space-occupying lesion with enhanced glycometabolism shown by PET-CT (red and white arrows).

As depicted in **Figures 1A, B**, a 57-year-old male was diagnosed with a primary hypolarynx squamous cell carcinoma (T2N1M0, stage III with a microinvasion) in July 2018 (t1). The patient was immediately subjected to a surgical resection to remove the malignant lesion. No abnormality was observed by abdominal color Doppler ultrasonography. From August 2018 (t2), the subject started receiving the intensity-modulated radiation therapy (IMRT, 60Gy/28F), concurrently in combination with three cycles of nedaplatin chemotherapy (140 mg) and six cycles of nimotuzumab targeted therapy.

**Figure 2:**
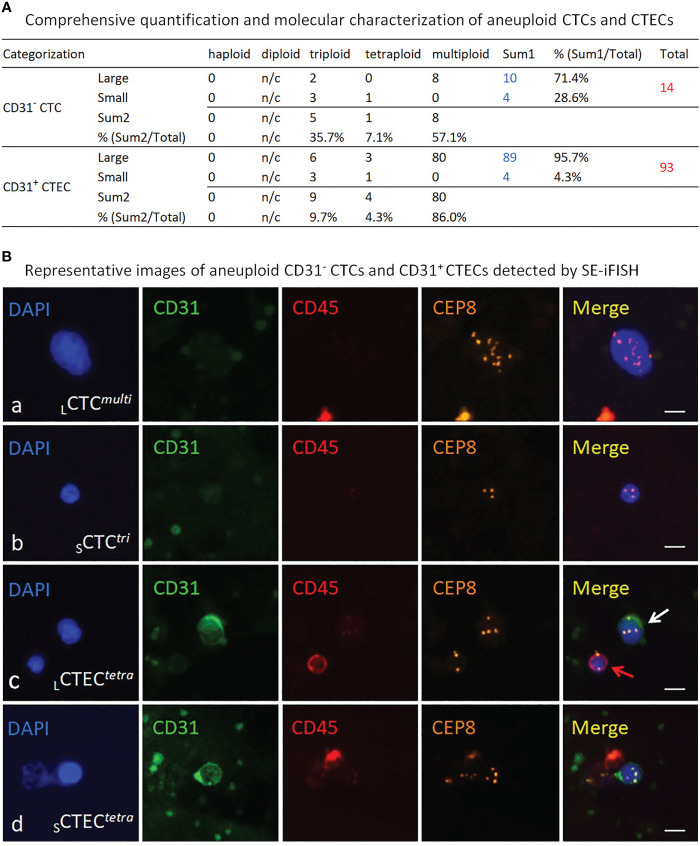
Detection and molecular characterization of aneuploid CD31^-^ CTCs and CD31^+^ CTECs performed by SE-iFISH. **(A)** Quantitative analysis of the detected CTCs and CTECs. Among 14 CTCs, 10 of them are large cells (10/14 = 71.4%), remaining 4 CTCs are in small cell size (28.6%). Different degrees of aneuploidy harbored by CTCs are trisomy 8 (35.7%), tetrasomy 8 (7.1%), and multisomy 8 (≥pentasomy 8, 57.1%), respectively. Most of detected 93 CTECs are large cells (89/93 = 95.7%), and the rest are small CTECs (4/93 = 4.3%). Degrees of aneuploidy in CTECs are 9.7% for triploidy, 4.3% for tetraploidy and 86% for multiploidy. n/c, not counted. **(B)** Representative images of the detected CTCs and CTECs. Ba, a large CTC with multiploid chr8 (CD31^-^/CD45^-^, >5 mm WBC size_, L_CTC*^multi^*). Bb, a small CTC with trisomy 8 (≤5 mm WBC_, S_CTC*^tri^*). Bc, a large tetraploid CTEC (CD31^+^/CD45^-^, _L_CTEC*^tetra^*) is indicated by a white arrow; a diploid CD45^+^ WBC is indicated by a red arrow. Bd, a small tetraploid CTEC (_S_CTEC*^tetra^*). Bars=5 μm.

Instead of longitudinal detection along with therapy process (15, 17, 22), co-detection of MRD aneuploid CD31^-^ CTCs and CD31^+^ CTECs was performed according to the protocol (13) at the end of combination regimens (t3, September 2018) in this case to evaluate surgery outcome and the therapeutic efficacy of the complete combination regimens. Comprehensive quantification and molecular characterization of aneuploid CTCs and CTECs were revealed in **Figure 2A**. A large quantity of 107 non-hematological circulating aneuploid rare cells including 14 CTCs and 93 CTECs were detected, with a high ratio of CTECs/CTCs > 5. Among 14 CD31^-^ CTCs, four were small sized cells (4/14 = 28.6%). A majority of CTCs were multiploid in chr8 (≥ pentasomy 8, 57.1%). Out of 93 CD31^+^ CTECs, most were large cells (89/93 = 95.7%). The predominant karyotype of CTECs was multiploidy. Large multiploid cells constitute the main population of detected CTECs (_L_CTECs*^multi^*, 80/93 = 86%). Representative images of CTCs and CTECs detected in this patient are demonstrated in **Figure 2B**.

In February 2019 (t4), as depicted in **Figures 1** **Ca, b**, the follow-up examination showed an increased cancer antigen 19-9 (CA19-9) (161.42 U/ml, reference range: 0-37 U/ml) and carcinoembryonic antigen (CEA) (6.71 ng/ml, reference range: 0-5 ng/ml) for the first time. However, the cervical MRI scan showed no laryngeal cancer recurrence. Additional abdominal color Doppler ultrasonography, abdominal CT scan (**Figures 1** **Da, b**), epigastric contrast enhanced (CE) MRI, and magnetic resonance cholangiopancreatography (MRCP) all showed no abnormality in pancreas and other visceral organs. The patient was diagnosed with a *de novo* pancreatic adenocarcinoma in July 2019 (t5). As illustrated in **Figures 1** **Ea–d**, epigastric MRI scan, CE-MRI, MRCP, and diffusion-weighted imaging (DWI) indicated the pancreatic head carcinoma. PET-CT in **Figures 1F, G** showed a pancreatic space-occupying lesion with enhanced glycometabolism.

The patient was subjected to four cycles of FOLFIRINOX chemotherapy in July, followed by palliative resection of the pancreatic lesion in September 2019 (t6) (Histopathology: moderately-to-poorly differentiated adenocarcinoma, 3*2*1 cm). The subject afterwards received seven cycles of FOLFIRINOX adjuvant chemotherapy. A follow-up examination of abdominal CT and MRI scan in March 2020 (t7) showed no additional tumor metastasis. The hepatic metastasis was diagnosed in July 2020 (t8). Following three cycles of Gem plus Taxotere (GT) chemotherapy, tumor progression was observed by abdominal MRI scan in August 2020 (t9). The patient was subjected to four cycles of FOLFIRINOX chemotherapy and subsequent combination therapy, whereas no progress was made on progression of the pancreatic cancer. The patient died in March 2021 (t10).

## Discussion

Liquid biopsy detection of circulating tumor biomarkers, including circulating tumor DNA (ctDNA) and CTCs, has served as a non-invasive approach applied in diagnosis and treatment of cancers, as well as detection of therapy-resistant MRD (24). ctDNA is composed of small DNA fragments secreted from tumor cells or the breakdown product of necrotic carcinoma cells including CTCs (25). Compared to the non-negligible drawbacks of the non-specificity of small fragment ctDNA due to its very low amount in blood (26), detection of viable and bioactive CTCs which possess aneuploidy (6, 27), cancer stemness (28), and epithelial-to-mesenchymal transition (EMT) (29) allows for real-time providing valuable clinical information more relevant to metastasis and therapeutic efficacy alongside the cancer treatment.

Aneuploidy is the hallmark of malignant neoplastic cells (6, 10, 27). The degree of aneuploidy is proportional to the grade of malignancy of carcinoma cells: the higher the degree of aneuploidy, the higher the malignancy grade, and the poorer the prognosis in cancer patients (30, 31). Moreover, aneuploidy was also reported to correlate with chemo- and targeted therapy resistance (22, 32). In addition to aneuploidy, recent studies on CTC morphology indicated that CTCs in small cell size (≤5 μm white blood cell) were relevant to gastric and lung cancer patients’ hepatic metastasis (33, 34), adverse prognosis (35), and post-surgical occurrence in hepatocellular carcinoma (HCC) patients (36). Unlike large cell CTCs, single cell DNA sequencing (scDNA-seq) analyses revealed that small cell sized CTCs exhibited a different therapy-resistance mechanism (33). Contrary to conventional CTC enumeration methods biased towards only EpCAM and CK-double positive neoplastic cells, which may bring a nonnegligible false negative detection due to intrinsic highly heterogeneities of cancer cells, SE-iFISH applied in this case allows to effectively co-detect, comprehensively characterize and subcategorize CTCs and CTECs into diverse subtypes based upon the degree of aneuploidy, tumor marker expression and cell morphology (cell size and cluster) (13, 21). Each subtype of CTCs and CTECs possess distinct clinical significance in terms of cancer metastasis, risk stratification, guiding targeted therapy, susceptibility or resistance to therapeutic agents, etc. (15, 17, 22, 23).

The present case showed that, after resection of the primary lesion and receiving a complete six cycles of chemoradiotherapy plus nimotuzumab, the patient still had a substantial amount of 107 CD45^-^ aneuploid circulating rare cells including 14 CTCs and 93 CTECs. Among those cells, 28.6% CTCs were in small cell size and 86% of CTECs as well as 57.1% CTCs had a high degree of chr8 aneuploidy (≥pentasomy 8). Comparing to an average of less than 2.8 ± 3.6 CTECs (Mean ± SD) per 6.5 ml blood in healthy donors (13), a large quantity of CTECs and CTCs detected in this patient after receiving a complete post-surgical chemoradiotherapy retrospectively suggested that the efficacy of the applied combination regimens was not as effective as expected.

It has been reported that CTCs do not exhibit the same chemosensitivity as the primary tumor cells in breast cancer patients (37). During a selective multistep metastasizing process, metastatic CTCs might be derived from only a few subclones of neoplastic cells in the primary lesion consisting of multiclonal carcinoma cells, followed by further hematogenous spreading selection in peripheral circulation. Eventually, survived CTCs and CTECs, which may have profound differences compared to primary tumor cells, participate in the formation of distant metastatic lesion(s). This may explain why CTCs and CTECs exhibit a different pattern of chemosensitivity to that of the primary tumor (38), indicating the necessity of active monitoring and characterization of CTCs and CTECs alongside and after treatment as for an adequate assessment of therapy effectiveness.

In addition to evaluation of therapeutic efficacy, the impact of CTCs on occurrence of malignancy was reported by others (2–5). In the current report, because squamous cell laryngeal carcinoma and the subsequent pancreatic adenocarcinoma histopathologically differ from each other, the pancreatic cancer was most likely the *de novo* malignancy following therapy. Post-therapeutically detected 107 aneuploid CTCs and CTECs were likely a mixture of laryngocarcinoma’s MRD (24), neoplastic cells from the subsequent *de novo* pancreatic cancer at the very early stage, and the new clones of dormant tumor cells awakened by the stress (39), all were resistant or insensitive to the applied combination regimens. CTCs were found as an effective indicator able to, despite invisible malignant lesions (40), independently predict early occurrence of pancreatic cancer (41) and melanoma in cancer patients (4). In non-small cell lung cancer (NSCLC) patients, CTCs positively detected one month after radial resection significantly correlated with an inferior prognosis and a high risk of early malignancy occurrence (7). As described in this report, two months after surgery, the patient showed the existence of abundant CTCs and CTECs upon finishing complete post-surgical combination therapy and, at the time, no abnormal blood test result or abnormal medical image scanning was observed. As depicted in **Figure 1**, positive detection of CTCs and CTECs (t3, August 2019) occurred five months prior to increasement of CA19-9 and CEA (t4, February 2019), and ten months prior to the diagnosed pancreatic cancer (t5, July 2019). Currently, most studies were performed to enumerate CTCs alone either at baseline or after surgery with regards to predicting cancer occurrence or recurrence, whereas the detection time point reported in this article is critical, indicating that detection of aneuploid CTCs and CTECs in a patient following receiving complete therapeutic regimens warrants an adequate evaluation of the finished treatments’ efficacy and an effective stratification of patient’s occurrence risk.

CTECs, harboring dual properties of both cancerous malignancy and endothelial vascularization ability, are predominately derived from the endothelialization of cancer cells either *in vivo* or *in vitro* (10, 42, 43), the process that enables carcinoma cells to express CD31 on their surface. CTECs, the CD31^+^ cancer cells in essence and referred to as a “wolf in sheep’s clothing” (10, 44), integrate multiple properties of epithelium, endothelium, mesenchyme, aneuploidy, malignancy, and motility. Thus, they are expected to play a vital role in tumorigenesis, neovascularization, disease progression, and cancer metastasis (10). We realized a large quantity and a high ratio of CTECs to CTCs > 5 in this case, and proposed for the first time that the large numbers and high ratio of CTECs/CTCs may associate with or impact cancers’ rapid occurrence.

Following recent progress in the innovation and clinical validation of tumor marker-iFISH, such as CA19-9/CEA/PD-L1/HER2-iFISH (10, 20), and the novel iFISH (NC) to distinguish and co-detect viable and necrotic aneuploid CD31^-^ CTCs as well as CD31^+^ CTECs (45), longitudinal monitoring of CTCs and CTECs with the updated six-channel multi-tumor marker-iFISH throughout combination regimens will help illustrate whether and how CTCs and CTECs expressing tumor markers have a mutual effect on tumor progression alongside treatment (15, 17, 22), thus allowing for a better assessment of therapy outcome and a more effective forecasting strategy of cancer relapse.

## Conclusions

Co-detection of aneuploid CD31^-^ CTCs and CD31^+^ CTECs in patients at the end of complete therapy cycles may provide a unique dual cellular evaluation and prediction approach, in terms of effectively evaluating therapeutic efficacy in post-therapeutic cancer patients, stratifying malignancy risk, and appropriately guiding systemic adjuvant therapy to target adequate subjects. A large amount and a high ratio of aneuploid CTECs to CTCs following a complete treatment indicate both the existence of therapy-resistant MRD and higher risk of cancer occurrence or metastatic distant recurrence. An effective therapeutic regimen, therefore, should be able to eradicate the targeted non-hematological aneuploid circulating rare cells in an effort to reduce the risk of post-therapeutic cancer occurrence or relapse (37).

## Data availability statement

The raw data supporting the conclusions of this article will be made available by the authors, without undue reservation.

## Ethics statement

The study was conducted according to the Declaration of Helsinki Principles. Informed consent form, approved by the Ethics Review Committees (ERC) of the Fifth Affiliated Hospital of Sun Yat-Sen University, Zhuhai, Guangdong, China, was signed and obtained from the patient.

## Author contributions

JM, FY, JL, and QZ participated in patient treatment and analysis of results. ML contributed writing original draft. AL contributed methodology and writing original draft. AL and DW contributed methodology and validation. QZ and PL contributed conceptualization, writing – original draft, review, and editing. All authors contributed to the article and approved the submitted version.

## Funding

This study was supported by the Science and Technology Planning Project for Health Care of Zhuhai City (General Project) Grant ZH22036201210051PWC to QZ.

## Acknowledgments

Authors thank staffs at the Fifth Affiliated Hospital of Sun Yat-Sen University, Cytointelligen (China Medical City, Taizhou, Jiangsu, China) and Cytelligen (San Diego, CA, USA) for providing support to this study.

## Conflict of interest

i•FISH^®^ is the registered trademarks of Cytelligen. PL is the president at Cytelligen. None of authors owns Cytelligen’s stock shares.

The remaining authors declare that the research was conducted in the absence of any commercial or financial relationships that could be construed as a potential conflict of interest.

## Publisher’s note

All claims expressed in this article are solely those of the authors and do not necessarily represent those of their affiliated organizations, or those of the publisher, the editors and the reviewers. Any product that may be evaluated in this article, or claim that may be made by its manufacturer, is not guaranteed or endorsed by the publisher.
